# Electric-Controlled Valley Pseudomagnetoresistance in Graphene with Y-Shaped Kekulé Lattice Distortion

**DOI:** 10.1186/s11671-020-3275-5

**Published:** 2020-02-19

**Authors:** Qing-Ping Wu, Lu-Lu Chang, Yu-Zeng Li, Zheng-Fang Liu, Xian-Bo Xiao

**Affiliations:** 1 Department of Applied Physics, East China Jiaotong University, Nanchang, 330013 China; 2 School of Computer Science, Jiangxi University of Traditional Chinese Medicine, Nanchang, 330004 China

**Keywords:** Pseudomagnetoresistance, Y-shaped Kekulé lattice, Graphene

## Abstract

We propose a new method for regulating valley pseudomagnetoresistance in ballistic graphene-based valley field-effect transistors by taking into account the Y-shaped Kekulé lattice distortion and electric barrier. The device involves valley injection and valley detection by ferromagnetic-strain source and drain. The valley manipulation in the channel is achieved via the Y-shaped Kekulé lattice distortion and electric barrier. The central mechanism of these devices lies on Y-shaped Kekulé lattice distortion in graphene can induce a valley precession, thus controlling the valley orientation of channel electrons and hence the current collected at the drain. We found that the tuning external bias voltage makes the valley pseudomagnetoresistance oscillate between positive and negative values and colossal tunneling valley pseudomagnetoresistance of over 30,000% can be achieved. Our results suggest that the synergy of valleytronics and digital logics may provide new paradigms for valleytronic-based information processing and reversible computing.

## Introduction

Graphene, being a two-dimensional sheet of carbon atoms, which have excellent carrier mobility and offers the thinnest possible channel for utilizing to design of metal-oxide-semiconductor field-effect transistors [1]. Semenov have proposed a spin field-effect transistor by utilizing a graphene layer as the channel [2], which involves spin injection and spin detection by ferromagnetic source and drain, and the spin manipulation in the channel is achieved via electrical control of the electron exchange interaction with a ferromagnetic gate. In addition, Rashba spin-orbit interaction is another promising tool for the spin control in graphene [3]. The Rashba spin-orbit interaction can induces a spin precession, thus controlling the spin orientation of channel electrons. The spin field-effect transistors also inspired many important research ideas, such as giant magnetoresistance and tunnel magnetoresistance [3, 4]. The giant magnetoresistance and tunnel magnetoresistance can be applied in digital storage and magnetic sensor technologies.

On the other hand, Dirac electrons in graphene possess extra valley degree of freedom besides the conventional charge and spin counterparts. Owing to the large momentum difference between the two valleys and the suppression of the intervalley scattering in clean graphene samples [5–7], the valley degree of freedom is believed to exert the same effect as the electron spin in carrying and manipulating information, which leads to a new discipline rising as valleytronics. In analog of spin field-effect transistor, valley field-effect transistor is also theoretically proposed in graphene [8], which consists of a quantum one dimension channel of gapped graphene sandwiched between two armchair graphene nanoribbons (source and drain); then, side gate electric field is applied to the channel and modulates the valley polarization of carriers due to the valley-orbit interaction, thus controlling the amount of current collected at the drain. However, due to the fact that the valley coupling in graphene has not become a physical reality for a long time, there are few further studies based on the valley field-effect transistors of graphene and related studies. Recent experiments by Gutierrez et al. [9] have revealed an unusual Y-shaped Kekulé(Kek-Y) bond texture in the honeycomb lattice on a graphene-copper superlattice, where one of six carbon atoms in each superlattice unit cell has no copper atoms below it and acquires a shorter nearest-neighbor bond. Further, Gamayun has shown that the Kek-Y bond texture offers a way for a momentum-controlled valley precession [10]. Beenakker et al. [11] showed that the Kek system can bring out a valley flip effect via the Andreev-like reflection. Rencently Wang et al. [12] found that the C-C bond-length modulation of the Kekulé lattice that keeps the inversion symmetry of the system can be used to manipulate the valley degree of freedom in a similar way to the exchange field precessing spin. This makes it possible to design a new type of valley field-effect transistor in graphene. Moreover, there is no report on the combined effects of the Kek-Y lattice distortion on the valley pseudomagnetoresistance in graphene. Valley pseudomagnetoresistance [13, 14] is analogous to the magnetoresistance in magnetic tunnel junction [15] where the magnitude of the spin current depends on the magnetic orientation of the electrodes [4].

## Methods

**Fig. 1:**
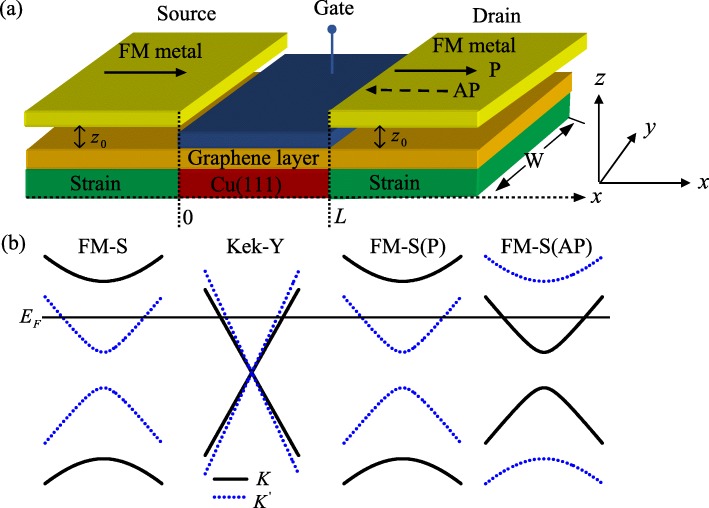
**a** Schematic illustration of the VFET utilizing a graphene channel with Kek-Y lattice distortion and a gate bias, which controls the valley orientation of channel electrons. The source and drain are FM-S graphene, which inject and detect electrons in a specific polarization. Where *z*_0_ is the distance between the graphene layer and the FM stripe. *L* is the channel length, *W* is the width of the graphene sample in the *y* direction, and *W*≫*L*. **b** Band structure near Dirac points. The horizontal line denotes the Fermi energy (color online)

In this work, we propose a new type of valley field-effect transistors (VFETs) for graphene-based electron. The device design assumes a ferromagnetic-strain (FM-S) source/drain for valley polarized injection/detection, which resembles conventional spin transistor (see Fig. 1a). Valley rotation in the graphene channel relies on Kek-Y graphene superlattice [10–12], which can be achieved by a superlattice of graphene grown epitaxially onto Cu(111), with the copper atoms in registry with the carbon atoms [9]. However, copper atoms are lacking under some carbon atoms, resulting in some periodic copper atom vacancies appearing below graphene. Such substrate atom vacancy leads to three neighboring bonds being contracted. Here, we use *δt* to represent the energy modification to the electron’s hopping corresponding to these three bonds. We assume that the ferromagnetic graphene is made of the same FM metal stripe. The two magnetizations of the source and drain are directed along the current direction (the *x* axis), which can be in either the parallel (P) or the antiparallel (AP) alignment, with the help of an external in-plane magnetic field. In the Landau gauge, the magnetic vector potential arising from the fringe field has the form [16, 17] $A(r)=A_{y}(x)\overrightarrow {y}$ with *A*_*y*_(*x*)=*A*_*y*_[*Θ*(−*x*)±*Θ*(*x*−*L*)], where the plus(minus) sign corresponds to the P(AP) configuration of magnetizations, *Θ*(*x*) is the Heaviside step function. On the other hand, we assume that the same strain are applied on source and drain of the VFETs, which can be induced by a tension on the substrate of the graphene [18]. The elastic deformation can be treated as a perturbation to the hopping amplitudes and acts as a gauge potential *A*_*S*_(*r*). The tension is set along the *x* direction, in this case, *A*_*S*_(*r*) uniform along the *y* axis [16]. For definiteness, we take a typical smooth profile of its *y* component as *A*_*Sy*_(*x*)=*A*_*S*_[*Θ*(−*x*)+*Θ*(*x*−*L*)], where *A*_*S*_ is the amplitude. In addition, a electic barrier are also applied in the Kek-Y lattice region, which can be tuned by external bias voltage.

The low-energy excitation quasiparticles propagation in the VFETs with Kek-Y graphene superlattices can be described by thes following single particle Hamiltonian [10–12]

1 $$ \begin{array} [c]{ll} H= & v_{F}(\mathbf{P}\cdot\sigma)+v_{\tau}(\mathbf{P}\cdot\tau)\Theta\left(x\right) \Theta\left(L-x\right) +\\ & U\sigma_{0}\tau_{0}\Theta\left(x\right) \Theta\left(L-x\right) +A_{M}(x)\sigma_{y}+\tau_{z}A_{S}(x)\sigma_{y}. \end{array}   $$

Here, *σ* and *τ* are the Pauli matrices for the sublattice and the valley, respectively. **P**=(*p*_*x*_,*p*_*y*_) is the momentum of massless Dirac electrons, *τ*_*z*_=±1 for *K* and $K^{^{\prime }}$ valleys, *v*_*F*_=10^6^m/s is the velocity of Dirac electrons in the pristine graphene, and *v*_*τ*_≃*v*_*F*_*δt*/3*t* is the velocity modification term from the bond contraction effect in the Kek-Y lattice [12], where *t* is the hopping energy between the nearest neighboring cites for pristine graphene. *U* is the gate-tunable potential barrier. *A*_*M*_(*x*)=*ev*_*F*_*A*_*y*_(*x*) [19]. The eigenvalues of the Hamiltonian in the graphene with Kek-Y lattice distortion and electric barrier are given by

2 $$ E_{\alpha,\beta}=U+\alpha(\hbar v_{F}+\beta\hbar v_{\tau})\sqrt{k_{x\beta} ^{2}+k_{y}^{2}}.   $$

Here, *α*=+1(−1) specifies the conduction (valence) band. *β*=±1 denotes the two valley-split subbands of the conduction and valence bands. Due to the translational invariance in the *y* direction, the transverse wave vector *k*_*y*_ is conserved. The eigenstates in the graphene with the homogeneous Kek-Y lattice distortion are characterized by $\Psi _{\beta }^{\pm }(k_{x\beta },k_{y})=\frac {1}{N_{\beta }}\left (1,P_{\beta }^{\pm },Q_{\beta } ^{\pm },R_{\beta }^{\pm }\right)^{T}$, where *N*_*β*_ is the normalization constant $N_{\beta }=\left (1+P_{\beta }^{2}+Q_{\beta }^{2}+R_{\beta }^{2}\right)^{\frac {1}{2}}$ and $P_{\beta }^{\pm }, Q_{\beta }^{\pm }$, and $R_{\beta }^{\pm }$ are functions defined as follows:

3 $$ \begin{array} [c]{cc} P_{\beta}^{\pm}= & \frac{(E-U)^{2}+\left(\hbar^{2}v_{F}^{2}-\hbar^{2}v_{\tau} ^{2}\right)\left(k_{x\beta}^{2}+k_{y}^{2}\right)}{2(E-U)\hbar v_{F}(\pm k_{x\beta}-{ik}_{y})},\\ Q_{\beta}^{\pm}= & \frac{(E-U)^{2}-\left(\hbar^{2}v_{F}^{2}-\hbar^{2}v_{\tau} ^{2}\right)\left(k_{x\beta}^{2}+k_{y}^{2}\right)}{2(E-U)\hbar v_{\tau}(\pm k_{x\beta}-{ik}_{y})},\\ R_{\beta}^{\pm}= & \frac{(E-U)^{2}-\left(\hbar^{2}v_{F}^{2}+\hbar^{2}v_{\tau} ^{2}\right)\left(k_{x\beta}^{2}+k_{y}^{2}\right)}{2\hbar^{2}v_{F}v_{\tau}(\pm k_{x\beta} -{ik}_{y})^{2}}. \end{array}   $$

The transmission probability from $K^{^{\prime }}$ valley to $K(K^{^{\prime }})$ valley $T_{K^{^{\prime }},K(K^{^{\prime }})}$ can be calculated using the transfer matrix technique [20]. According to the Laudauer-Btittiker formula, the valley-dependent conductance is given by [21]:

4 $$ G_{K^{^{\prime}},K(K^{^{\prime}})}=G_{0} {\int_{-\frac{\pi}{2}}^{\frac{\pi}{2}}} T_{K^{^{\prime}},K(K^{^{\prime}})}\cos(\phi_{0})d\phi_{0}.   $$

Here $G_{0}=2e^{2}W/\left (v_{F}\pi ^{2}\hbar ^{2}\right)\left \vert E\right \vert $, *W* is the width of the graphene sample in the *y* direction, and *ϕ*_0_ is the incident angle with respect to the *x* direction.

Before proceeding with the calculations, we discuss the band structure with *k*_*y*_=0, as shown in Fig. 1b. In the FM-S source region, the energy band of graphene is written as $E=\alpha \sqrt {(\hbar v_{F}k_{x})^{2} +(A_{M}+\tau _{z}A_{S})^{2}}$. One can find that the valley degenerate is lift and different gaps are induced at the *K* and $K^{^{\prime }}$ points because the total vector potential *A*_*M*_+*A*_*S*_ acting on *K* electrons is higher than the total vector potential |*A*_*M*_−*A*_*S*_| acting on for $K^{^{\prime }}$ electrons [19]. This indicates that only $K^{^{\prime }}$ electrons can pass through the FM-S source region when the incident energy is located in |*A*_*M*_−*A*_*S*_|<*E*<*A*_*M*_+*A*_*S*_ [22, 23]. Similarly, in the FM-S drain region, the energy band of graphene can be written as $E=\alpha \sqrt {(\hbar v_{F}k_{x})^{2}+(\pm A_{M}+\tau _{z}A_{S})^{2}}$, where the ± sign corresponds to the P and AP configuration of magnetizations. So only $K^{^{\prime }}$ electrons are detected in the P structure and only *K* electrons are detected in the AP structure when the Fermi energy locates at the range of [|*A*_*M*_−*A*_*S*_|,*A*_*M*_+*A*_*S*_]. In the graphene channel, the valley degenerate is also lift, but there is an important difference. In contrast to the lead case, where the phases of *K* and $K^{^{\prime }}$ components evolve with the same wave vector [i.e., $k=E/\hbar v_{F}$], now, they evolve separately with different wave vectors ($k_{+}=(E-U)/(\hbar v_{F}+\hbar v_{\tau })$ and $k_{-}=(E-U)/(\hbar v_{F}-\hbar v_{\tau })$) due to the Kek-Y graphene superlattices mixing the valley (see Eq. 2). This leads to the valley precession of channel electrons in the valley space [12]. The valley precession in graphene is the basis for the valley field effect transistor [8]. And the valley precession can also be characterized by a valley pseudomagnetoresistance (VPMR) in the FM-S/Kek-Y/FM-S junctions, analogous to the magnetoresistance in graphene-based quantum tunneling junctions with the spin-orbit interaction [4], which is defined as $VPMR=\frac {G_{P}-G_{AP}}{G_{P}}$, where *G*_*P*_ and *G*_*AP*_ represent the conductance in P and AP configurations, respectively, and $G_{P}=G_{K^{^{\prime }},K^{^{\prime }}}, G_{AP}=G_{K^{^{\prime }},K}$. The magnitude of the valley current depends on the magnetic orientation of the source and drain in our considered device.

## Numerical Results and Discussions

**Fig. 2:**
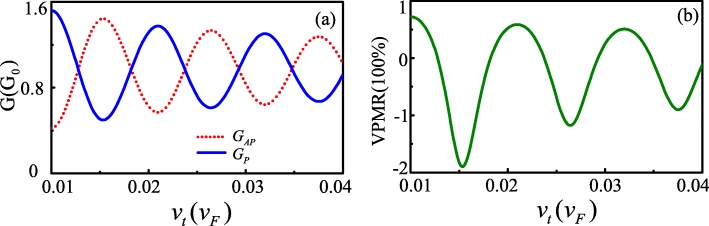
Conductance *G*_*P*,*AP*_ and VPMR versus *v*_*t*_ at *L*=207nm,*E*=80meV and *U*=−10meV (color online)

In the following, we present the numerical results for the FM-S/Kek-Y/FM-S junction in graphene. Throughout the paper, we set channel length *L*=207nm, and restrict the Fermi energy 20 meV<*E*<140meV, assumed it satisfying |*A*_*M*_−*A*_*S*_|<*E*<*A*_*M*_+*A*_*S*_. Figure 2a and b show the calculated results of tunneling conductance and VPMR as a function of *v*_*t*_ with Fermi energy *E*=80meV and rectangle potential barrier *U*=−10meV. We can find that *G*_*P*_ and *G*_*AP*_ have the same oscillation periods but the inverse phases. Therefore, the VPMR oscillates with increase of *v*_*t*_ and the negative value VPMR can appear. Those phenomena are similar to the case of the magnetoresistance in ballistic graphene-based quantum tunneling junctions with the spin-orbit interaction [4]. The oscillation characters of the conductance of *G*_*P*_ and *G*_*AP*_ can be explained by the phase difference between the two valley components. When the incident angle *ϕ*_0_=0, the phase shift is given by: $\Delta \theta =(k_{x+}-k_{x-})L=-\frac {2(E-U)v_{\tau }}{\hbar (v_{F}^{2}-v_{\tau }^{2})}L$. *Δθ* determines the orientation of valley polarization before the electron enters the drain, relative to that of the drain state [8]. For *Δθ*=±2*nπ*,*n*=1,2,3⋯, the two polarizations are aligned, leading to the conductance *G*_*P*_ maximum and VPMR a high positive value (as seen in *v*_*τ*_=0.022, 0.033). On the other hand, for *Δθ*=±(2*n*+1)*π*,*n*=0,1,2⋯, they are orthogonal to each other, leading to the conductance *G*_*AP*_ minimum and VPMR negative (as seen in *v*_*τ*_=0.0167, 0.027, 0.038).

**Fig. 3:**
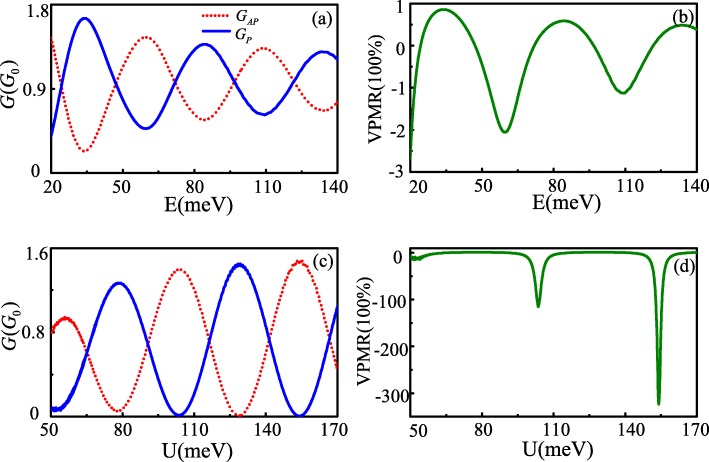
Conductance *G*_*P*,*AP*_ (**a**, **c**) and VPMR (**b**, **d**) as functions of the Fermi energy and the electric barrier at *L*=207nm,*v*_*t*_=0.02*v*_*f*_. the other parameters are *U*=−10meV for **a** and **c**, *E*=80meV for **b** and **d** (color online)

The conductance and VPMR are not only oscillation functions of the hopping-energy modification, they also oscillate with Fermi energy and the effective barrier potential since *Δθ* scales are also linear with the Fermi energy and the potential barrier *U*. Figure 3a and b show the conductance as a function of Fermi energy and the effective barrier potential, respectively. The corresponding VPMR are given in Fig. 3c and d. They all show oscillation characteristics varying with *E* and *U* value, even when the effective barrier potential *U* is greater than Fermi energy *E*. The physical origin for such a phenomenon is related to the Klein tunneling [12]. Although there are similar oscillation phenomena of conductance and VPMR for increased *E* and *U*, some differences can be also found. As *E* increases, the difference between *G*_*P*_ and *G*_*AP*_ conductance becomes smaller and smaller, which lead the oscillation amplitude of VPMR to becomes decreased with the increase of Fermi energy. While under the condition *Δθ*=±*nπ* is satisfied, the difference between *G*_*P*_ and *G*_*AP*_ is greater with increasing of *U*, especially in some location, the *G*_*P*_ and *G*_*AP*_ conductance presents switching characteristics. The characters are more desirable for the application of VPMR. Remarkably, the observed maximum value of VPMR is over 30,000% at small *E*. This value greatly exceeds MR of *~* 175*%* in the ballistic graphene-based quantum tunneling junctions with the spin-orbit interaction [4] and the pseudomagnetoresistance of *~* 100*%* in bilayer graphene controlled by external gates [24], which is even larger than the VPMR of *~* 10000*%* in a merging Dirac cones system [13].

## Conclusions

In conclusion, we proposed a type of valley field-effect transistors for graphene-based electron and studied the valley pseudomagnetoresistance through it. We have shown that the oscillation feature of valley pseudomagnetoresistance not only related to the hopping-energy modification and Fermi energy, but also can be tuned largely by the effective barrier potential. The valley pseudomagnetoresistance tuned by external bias voltage benefits the valley field-effect transistor device, and we anticipate that the electric controlled valley quantum devices proposed here can play a role in quantum and quantum-classical hybrid computers.

Further research could involve the different strain (uniaxial vs. biaxial) tunable the valley scattering of electrons and transport in our proposed graphene-based valley field-effect transistors since the stain is useful to control the degree of intervalley scattering in Kekulé patterns [25]. Then, other two-dimensional materials (MoS_2_, WS_2_, WSe_2_, etc.) analogs in graphene can also provide an interesting platform for other two-dimensional material-based valley field-effect transistors with Y-shaped Kekulé lattice distortion.

## Data Availability

The datasets supporting the conclusions of this article are included within the article.

## Abbreviations

AP: Antiparallel
FM-S: Ferromagnetic-strain
Kek-Y: Y-shaped Kekulé
P: Parallel
VFETs: Valley field-effect transistors
VPMR: Valley pseudomagnetoresistance
